# Evaluation of a pilot, community-led mental illness de-stigmatization theater intervention in rural uganda

**DOI:** 10.1186/s12888-022-04441-w

**Published:** 2022-12-16

**Authors:** Yang Jae Lee, Kazungu Rauben, Curtis Liu, Rebecca Kim, Nina van der Velde, Chelsea Taylor, Alyssa Walsh, Mildred Asasira, Ivan Katongole, Jolee Hatfield-King, Scott Blackwell, Theddeus Iheanacho, Ryan Christ, Ibrahim Ssekalo

**Affiliations:** 1 Yale University, New Haven, USA; 2 Empowerment to Heal - Uganda, Kampala, Uganda; 3 Williams College, Williamstown, USA; 4 The Ohio State University, Columbus, USA; 5 Uganda Christian University, Mukono, Uganda; 6 Empower Through Health, St. Louis, USA

**Keywords:** Mental health, Stigma, Art, Theater, Narrative, Psychiatric disorders, Schizophrenia, Psychosis, Mental illness, Community-led interventions

## Abstract

**Background:**

In rural areas of low- and middle- income countries, mental health care is often unavailable and inaccessible, and stigma is a major barrier to treatment. Destigmatization can increase treatment-seeking attitudes, community support, and acceptance of individuals suffering from mental illness. This study’s primary objective was to evaluate the impact of a community-led, theater-based destigmatization campaign for mental illness conducted in the Busoga region of Eastern Uganda.

**Methods:**

One hundred residents of the Busoga region were randomly selected via cluster sampling to complete a structured questionnaire assessing mental health stigma. Four focus groups were conducted for qualitative data on mental health stigma. Common misconceptions and specific points of stigma were identified from these responses, and local village health team personnel developed and performed a culturally-adapted theatrical performance addressing these points. Changes in perceptions of mental illness were measured among 57 attendees using two measures, the Broad Acceptance Scale (designed to reflect factors that contribute to structural stigma) and Personal Acceptance Scale (designed to reflect factors that contribute to interpersonal, or public stigma), before and after the performance.

**Results:**

There was a significant increase in acceptance according to the Broad Acceptance Scale (*p* < .001) and Personal Acceptance Scale (*p* < .001). Qualitative responses from play attendees also indicated a decrease in stigma and an increased sense of the importance of seeking treatment for mentally ill patients.

**Conclusion:**

This study shows community-led, theater intervention may be an effective tool for the destigmatization of mental illness in rural areas of Uganda. Larger studies are needed to further test the efficacy of this approach and potential for longer-term scalabilityand sustainability.

**Supplementary Information:**

The online version contains supplementary material available at 10.1186/s12888-022-04441-w.

## Background

Mental health is an essential and defining component of overall health that affects the ability of individuals to take care of themselves, maintain relationships with others, and perform activities necessary for daily living (ADLs) [1]. In low-income countries, the prevalence of significant stressors such as poverty, urbanization, and migration contributes to a high burden of mental illness. Mental health services are chronically underfunded in low-income countries; often less than 1% of the total healthcare budget is allocated toward mental health services [2]. Low-income countriess also commonly lack legislation to support community mental health care interventions and professionals, and insufficient infrastructure impedes access to these programs even when such policies do exist [2]. Thus a lack of adequate mental health resources, facilities, and health workers all compound to form significant roadblocks for those seeking care [3, 4]. The end result is an overburdened healthcare system that is unequipped to deal with the high prevalence of mental illness, causing individuals to rely primarily on their communities and family members to have their mental health needs met.

Because individuals suffering from mental illness in low-income countries are primarily dependent on members of their community for support, inaccurate or harmful beliefs surrounding these conditions can negatively impact health outcomes. Stigma-based discrimination against those with mental illness is a well-documented phenomenon. The presence of mental health stigma is associated with fewer employment opportunities, lower quality healthcare, increased poverty, and a reduction in help-seeking behavior for the affected individual [5]. These associated effects can result in homelessness, malnutrition, and increased stress, all of which have been shown to further exacerbate underlying mental illness [5]. Meta-analysis has shown repeatedly that stigma against those with mental illness in low-income countries is common, associated with lower quality of life, and leads to adverse health outcomes [6]. Due to the substantial role that stigma plays in discouraging the use of mental health resources, anti-stigma interventions are an essential part of any strategy that seeks to increase access to treatment and reduce the burden of mental illness.

Over the past few decades, strategies for destigmatization have centered around community education, protest campaigns, and direct engagement with those at risk [7]. Educational methods are the most common tool utilized by stigma reduction campaigns in low-income countries, however a surprisingly low number of creative-based interventions have been published in the literature [3]. Although theatrical performance has proven effective for destigmatizing HIV/AIDS in low-income countries, [8] little is known about how theater-based interventions affect attitudes around and stigma towards mental illness in low-income countries. However, in high-income counries, there is ample evidence that theater and film-based interventions reduce mental health stigma and enhance mental health literacy in the international literature [9, 10]. For example, a study showed that a stage performance targeting stigmatizing attitudes towards bipolar disorder produced immediate impact on reducing stigma quantitatively and qualitatively of healthcare providers [11]. In sub-Saharan Africa, theater-based approaches for health promotion have been the most common arts-form approach for health education [12]. Because such community-led interventions are culturally adapted, low-cost, and tailored to address specific stigmas within a community, they have been reported to have a high likelihood of success [8, 13]. One similar study investigated the feasibility of community-directed theater competitions to address stigma against psychosis in Zimbabwe [14]. However, despite promising results, the intervention’s impact on audience members’ mental health stigma was not directly measured; [14] to our knowledge, this is the first study attempting to quantify impact that theatrical performances may have on mental illness stigma in a rural community in a low-income country.

To assess the potential of culturally-specific theater programs to change community attitudes and reduce mental health stigma in low-income countries, we developed and pilot-tested a community-led performance in the Busoga region of eastern Uganda, which depicted an individual’s struggle with mental illness and journey to receive appropriate care. By showing this man’s progression from being severely debilitated and ostracized to becoming integrated as a productive member of society, we ascribed a face and voice to similar individuals whose suffering is very real and humanize their difficulties for the rest of the community. The efficacy of our intervention was measured by surveying audience members to ascertain personal and broad acceptance of individuals with mental illness both prior to and one week after viewing the theatrical production.

## Methods

### Study setting

**Fig. 1:**
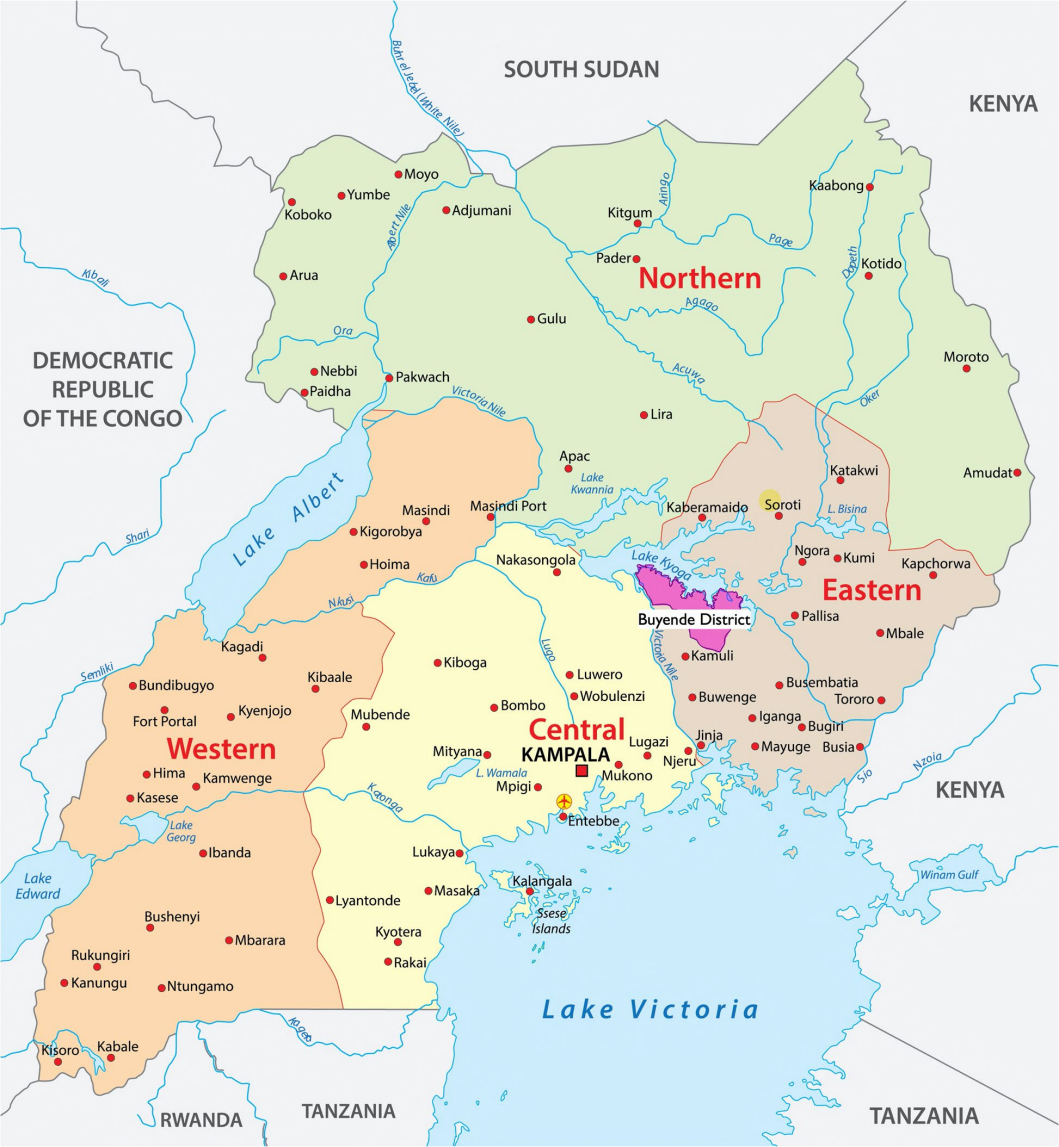
Location of Buyende District within the eastern region of Uganda. Regions of Uganda (Map from WorldAtlas) [17]

**Fig. 2:**
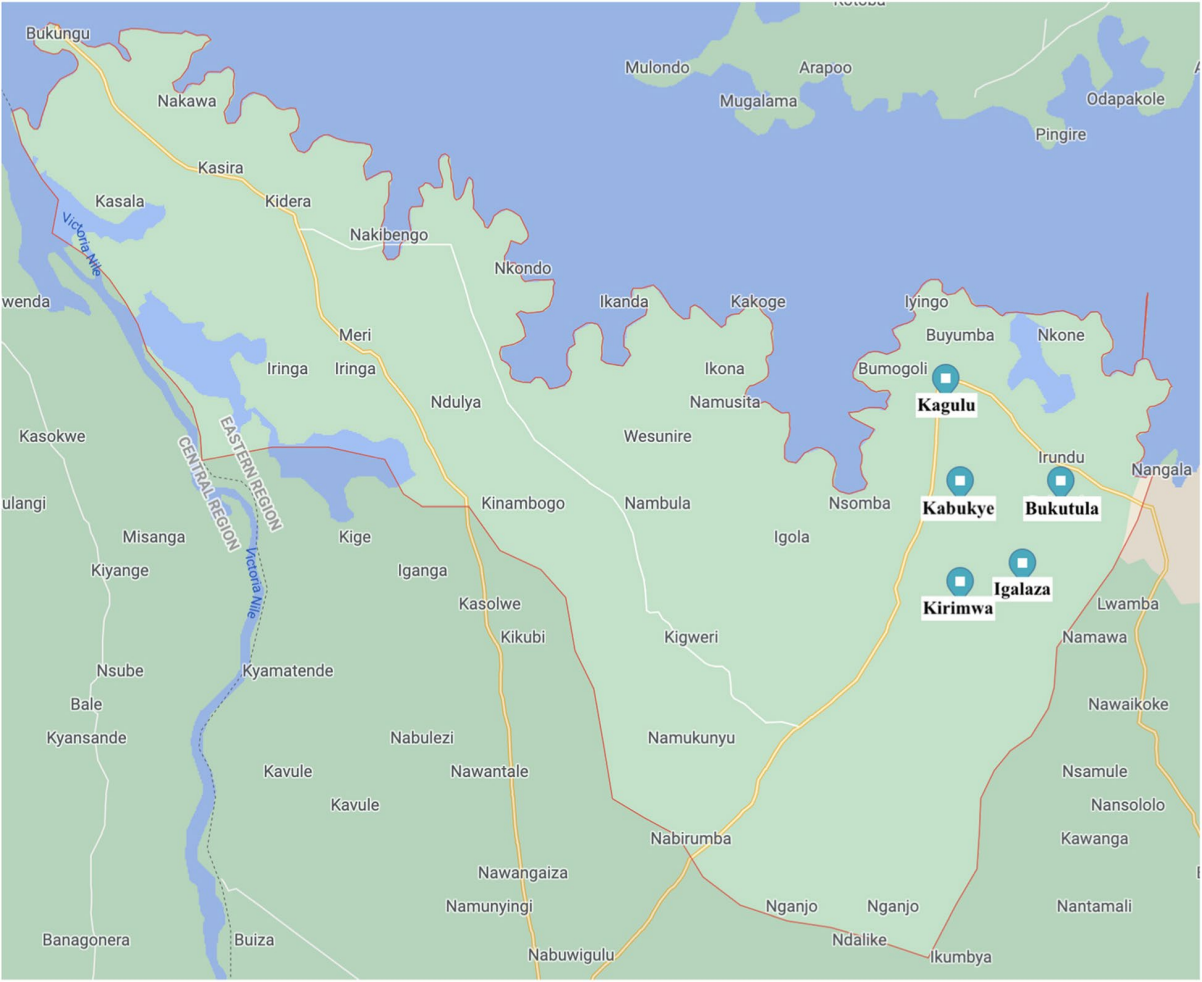
Parishes in Buyende District where the study was conducted (Map data ©2022 Google Maps) [18]

This study was conducted in Buyende District, a rural district in the Busoga region of eastern Uganda, which has a 2020 population projected at 414,600 [Fig. 1] [15]. Villages were randomly sampled from five out of Buyende’s sixty-eight parishes (Bukutula, Kabukye, Kagulu, Kirimwa, Igalaza) [Fig. 2] [16].

Buyende District was selected because it is representative of many rural areas in Uganda and other sub-Saharan African countries that do not have regular access to mental health services. About two-thirds of households are five kilometers or more from the nearest public health facility, and to our knowledge there is only one mental health professional operating in the entire district [15]. A majority of the population is under the age of 18, and 45% of individuals aged 18 and above are illiterate [15]. Most of the working population are subsistence farmers, and agriculture is a dominant economic sector in the region [15].

### Questionnaire design and administration

Alongside background demographic and socioeconomic questions, the 61-item questionnaire administered to an initial cohort of 101 participants included two custom batteries designed to measure the effectiveness of the community theater intervention program in reducing stigma towards people with mental illnesses: the Personal Acceptance Scale (9 questions) and the Broad Acceptance Scale (10 questions). Both scales were adapted from a study examining attitudes and beliefs about mental illness in Nigeria [19]. The original questionnaire utilized in the Nigerian study modified items taken from the Fear and Behavioral Intentions towards the mentally ill questionnaire, [20] selected items from the Community Attitudes to Mental Illness scale, [21] and a modified version of a questionnaire developed for the World Psychiatric Association: Program to Reduce Stigma and Discrimination [22].

Items included in the Personal Acceptance Scale targeted public stigma, the “negative attitudes, beliefs, and behaviors held within a community” against individuals with mental illness [23]. Items included in the Broad Acceptance Scale targeted structural stigma, the “societal-level conditions, cultural norms, and institutional practices that constrain the opportunities, resources, and wellbeing” for individuals with mental illness (See Table 1). We thought it was important to measure structural stigma separate from public stigma since structural stigma is often overlooked in the literature [24]. We did not attempt to measure self-stigma, the negative attitudes that people with mental illness have about their own condition, since our study did not explicitly survey individuals with mental illness [25].

The initial 61-item questionnaire was administered pre-intervention; post-intervention, only the two acceptance scales were administered, alongside a few additional open-ended questions about the intervention.

**Table 1:** Broad Acceptance Scale and Personal Acceptance Scale questions

Broad Acceptance Scale^a^	Personal Acceptance Scale
^b^ People with mental illness are a public nuisance	^b^ Are you afraid of people with mental illness?
^b^ Anyone with mental illness should not be given any responsibility	^b^ Would you object to having mentally ill people living in your neighborhood?
^b^ People with mental illness are a burden on society	Would you be willing to work with someone with a mental illness?
People with mental health problems should have the same rights to a job as anyone else	Would you invite someone into your home if you knew they suffered from mental illness?
We have a responsibility to provide the best possible care for people with mental illness	^b^ Would you not want to live next door to someone who has been mentally ill?
^b^ Increased spending on mental health services is a waste of money	^b^ It is frightening to think of people with mental problems being neighbors
We need to adopt a far more tolerant attitude toward people with mental illness in our society	Would you have casual conversations with neighbors who had suffered from mental illness?
Less emphasis should be placed on protecting the public from people with mental illness	Most women who were once patients in a mental hospital can be trusted to watch my child
^b^ Anyone with a history of mental problems should be excluded from taking public office	^b^ Would you avoid conversations with neighbors who had suffered from mental illness?
People with mental illness can work in regular jobs	

^a^Scales give a measure of broad or community-level acceptance towards people with mental illness. Questions had yes or no answers, and these responses were converted to binary 1 or 0 numbers. Numbers were summed and fit to a scale of 0–10

^b^Reverse scored

Each yes added one point to the scale, and five questions from each scale were reverse-scored to match the direction of the scale. The total number of points was divided by the number of questions (nine) and multiplied by ten to generate numbers between zero and ten for comparison purposes to the Broad Acceptance Scale. All questions were initially created in English, translated into Lusoga by a native speaker, and then back-translated by a different native speaker in order to ensure that the meaning was intact when the survey was administered. The initial set of questions was administered as a trial run in a village that was not a part of the study to determine efficacy and understanding. Questions and translations were then modified as needed.

Once the initial participants, referred to as the baseline cohort, were selected, a research assistant fluent in both Lusoga and English administered the pre-intervention survey in the field, relaying the answers to a data recorder who input results into the KoBoToolbox software on a password-protected mobile device and sent data to an online repository. Before the survey, research assistants read a consent form describing the aim of the study and any potential risks and discomforts to request verbal and written consent. The consent forms were translated into Lusoga by native speakers and back-translated by a fluent Lusoga-speaking psychiatric clinical officer to ensure accuracy. In the case that participants were illiterate or wary of signing any written documents, an impartial witness was asked to sign the consent form.

### Theater play design

Development of the theatrical production to destigmatize mental illness guided by the pre-intervention questionnaire responses as well as focus group discussions. Guidelines and instructions were distributed to community health workers in Buyende District (Appendix A). Community health workers, sometimes also known as village health teams in Uganda, are community-selected volunteers who are responsible for conducting home visits, distributing health commodities, and referring people to health facilities [26]. They are a trusted primary health contact for community members and their involvement in the study was intended to establish trust and credibility with the community. Because initial focus groups and community health workers considered psychosis as the most pertinent mental illness, the community participants were asked only to illustrate psychosis, excluding other common mental illnesses such as depression. A competition among the four groups of community participants was held, after which the community health workers voted to determine which group best met the criteria and created the most compelling, informative performance.

The winning group from Kabukye Parish held five performances of their play during a one-week period for the baseline cohort and other community members who came to watch, occurring approximately six weeks after the pre-intervention survey. The winning group portrayed a man with psychosis who was first ostracized from the community. He was then taken to different locations where he could seek help. He first went to the traditional healer where they conducted rituals including animal sacrifices and exorcisms, which were not effective. Then, he went to a religious center where they conducted a prayer and a song, which also was ineffective. He then went to the health center where he received medications and after several weeks, he returns as a motorcycle taxi driver, which is a well regarded profession in a rural setting in Uganda. Additionally, research assistants and a licensed psychiatric clinical officer presented a brief verbal training after each performance that specifically addressed stigmatized points identified by the pre-intervention survey and focus groups to encourage the audience to seek treatment for mental illness at their local health center. Those of the baseline cohort who had attended the play were then evaluated with the post-intervention survey approximately one week after the intervention to determine its impact on individuals’ acceptance of those with mental illness. The post-intervention survey consisted of the same questions as the previous survey as well as a few additional open-ended questions about the intervention.

### Focus Group Design

Focus groups with community members selected through convenience sampling were conducted to assess qualitative data about stigma towards mental health before the intervention. Seven focus group questions were designed to inquire into similar themes as the questionnaire and were translated and back-translated in the same manner. A trial focus group was conducted to screen the focus group questions for thematic clarity and comprehensibility: the focus group discussion was recorded and transcribed, then translated into English to allow for needed modifications to the focus group questions to be made. The questions were then translated back into Lusoga before proceeding with four official focus group discussions for data collection. Each focus group lasted between thirty minutes and one hour and consisted of between six and ten participants. Each focus group was recorded with the consent of the individuals involved. Recordings were subsequently transcribed and responses grouped into thematic areas where stigma seemed to be concentrated. Codebook with themes, sub-themes, and illustrative quotes can be found in Appendix D.

### Selection of participants

The baseline cohort was recruited from the Buyende District using stratified sampling. Ten villages in the Buyende District were randomly selected for participation in the study: all villages in the district were assigned a number; ten numbers were randomly selected, and a list of the corresponding villages was created. Every house in each village was assigned a number using census data recorded by the village’s community health worker. The same number assignment and random selection process were used with this census to create a list of ten households to be surveyed. At each selected household, researchers obtained a list of every household member aged 18 years or older; these individuals were assigned a number and one was randomly selected. If the selected individual was not home when researchers visited, a different individual from the same household was selected using the same process of random selection. If no household members fit the above inclusion criteria, a different household was randomly selected. This process was iterated until ten participants were recruited from each village. For focus groups, participants were selected by a community health worker based on location and availability. Each focus group contained roughly six to ten participants, stratified by gender and age to accurately represent a cross-section of the adult population. Survey participants were told that participation was optional and that they would not be monetarily reimbursed for their time. However, focus group participants were given a small stipend for their time and effort. After the performance of the winning play, the initial 101 participants of the baseline cohort were contacted via telephone to ensure that they had watched the play. Those that had watched the play were asked if they were available for a follow-up in-person survey within a week. Out of the 101 initial participants, 77 attended the performance, and 57 completed the post-intervention survey.

### Inclusion criteria

All individuals at the community level in the selected study site over 18 years old and able to provide informed consent were eligible for the study.

### Exclusion criteria

Individuals under the age of 18 or who could not give consent to participate in this study were excluded.

### Cohorts

Baseline cohort: the 101 randomly selected participants who were administered the pre-intervention survey. Intervention cohort: the 57 participants who responded to the pre-intervention survey, attended the play, and responded to the post-intervention survey. Pre- and post-intervention responses: responses from the intervention cohort in the pre- and post-intervention surveys, respectively.

### Participant information

Thirteen males and 44 females comprised the 57 participants in the intervention cohort. Their average age was 38.7 years old, and most participants were peasant farmers (48 out of 57). 47 participants were Christian (Pentecostal, Catholic, or Anglican), and 10 were Muslim. 3 participants had completed their O-levels, 17 had completed primary school, and the remaining 37 had either not completed primary school or never attended. These proportions were comparable to those of the baseline cohort (Appendix B, Table B1).

### Statistical method

Out of the survey questions, nineteen were grouped thematically into two scales, the Broad Acceptance Scale (10 questions) and the Personal Acceptance Scale (9 questions). The two scales indicated two overarching categories of acceptance towards people with mental illness: either broad, community, and abstract acceptance, or personal attitudes and opinions (See Table 1). Categorical yes-no answers to the pre- and post-intervention survey questions were converted to 1 or 0 respectively and reverse scored as necessary so increased scores on the scales indicated greater acceptance and less stigma. Both scales were rescaled to range from 0 to 10, and paired samples t-tests were run for each scale and each survey question individually. The focus groups were analyzed using constant comparison analysis to compare the results from multiple focus groups [28].

## Results

**Fig. 3:**
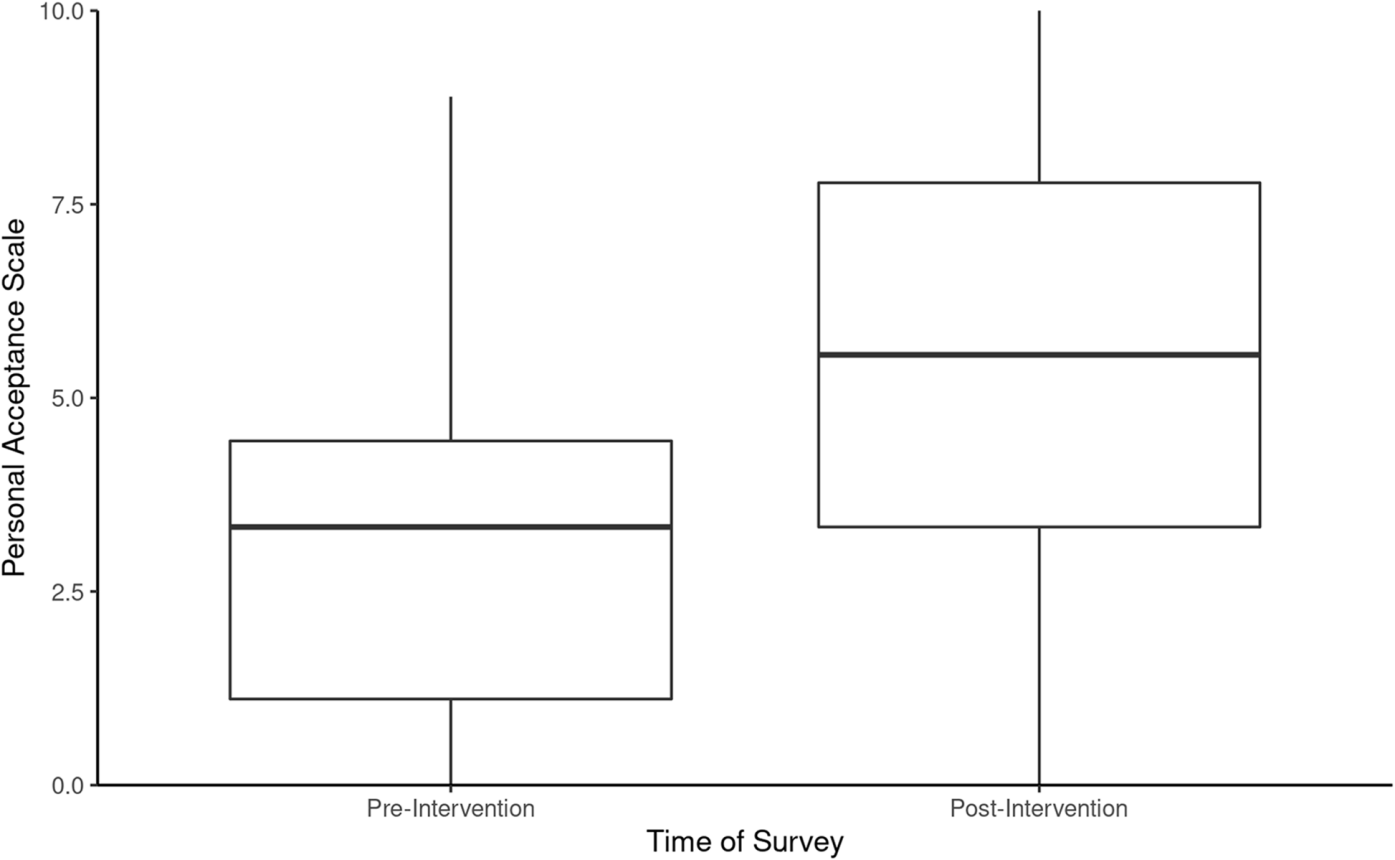
Personal Acceptance Scale values before and after the intervention (*n* = 57). The Personal Acceptance Scale ranged from 0 (lowest personal acceptance) to 10 (highest personal acceptance)

**Fig. 4:**
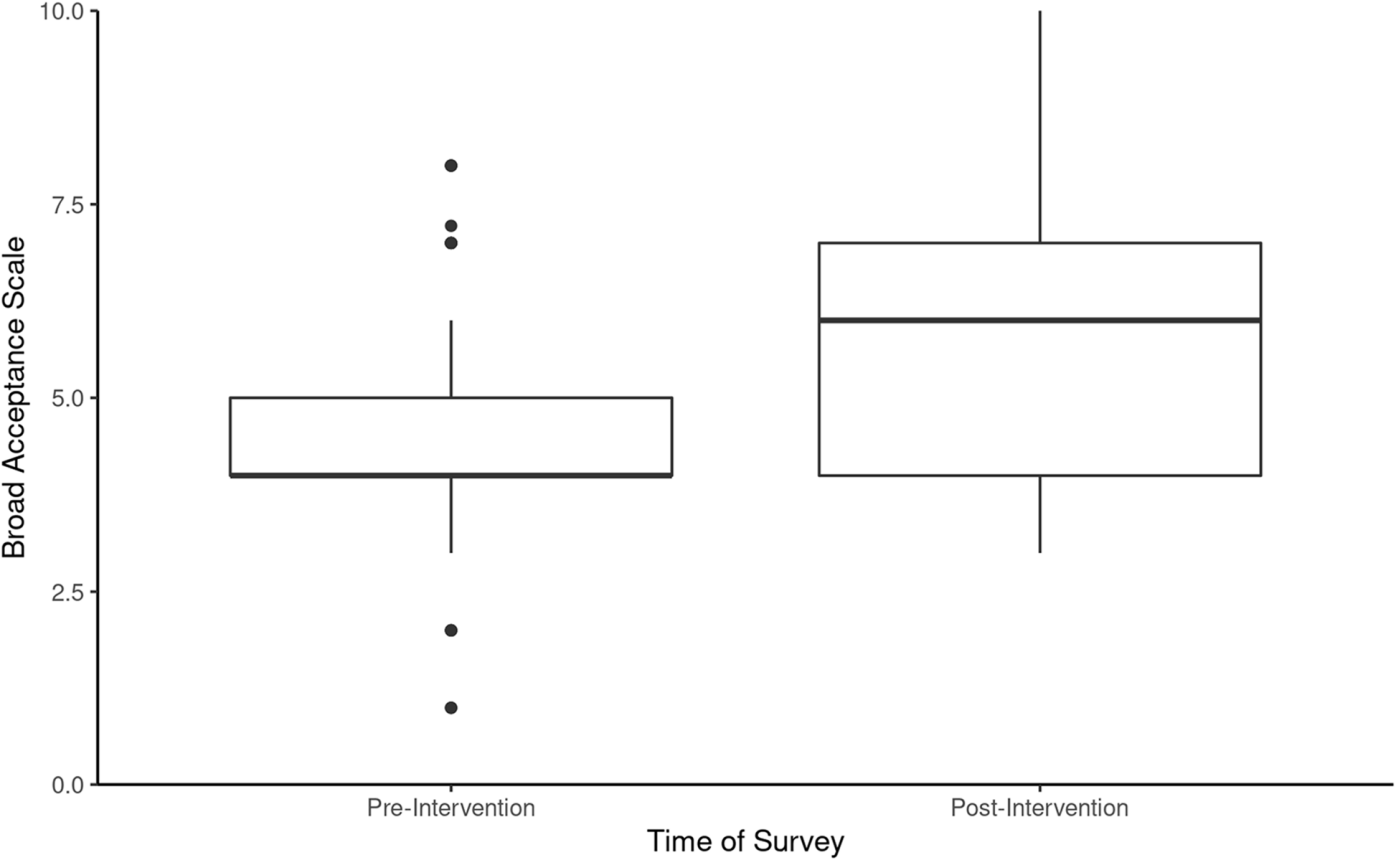
Broad Acceptance Scale values before and after the intervention (n = 57). The Broad Acceptance Scale ranged from 0 (lowest broad acceptance) to 10 (highest broad acceptance)

**Fig. 5:**
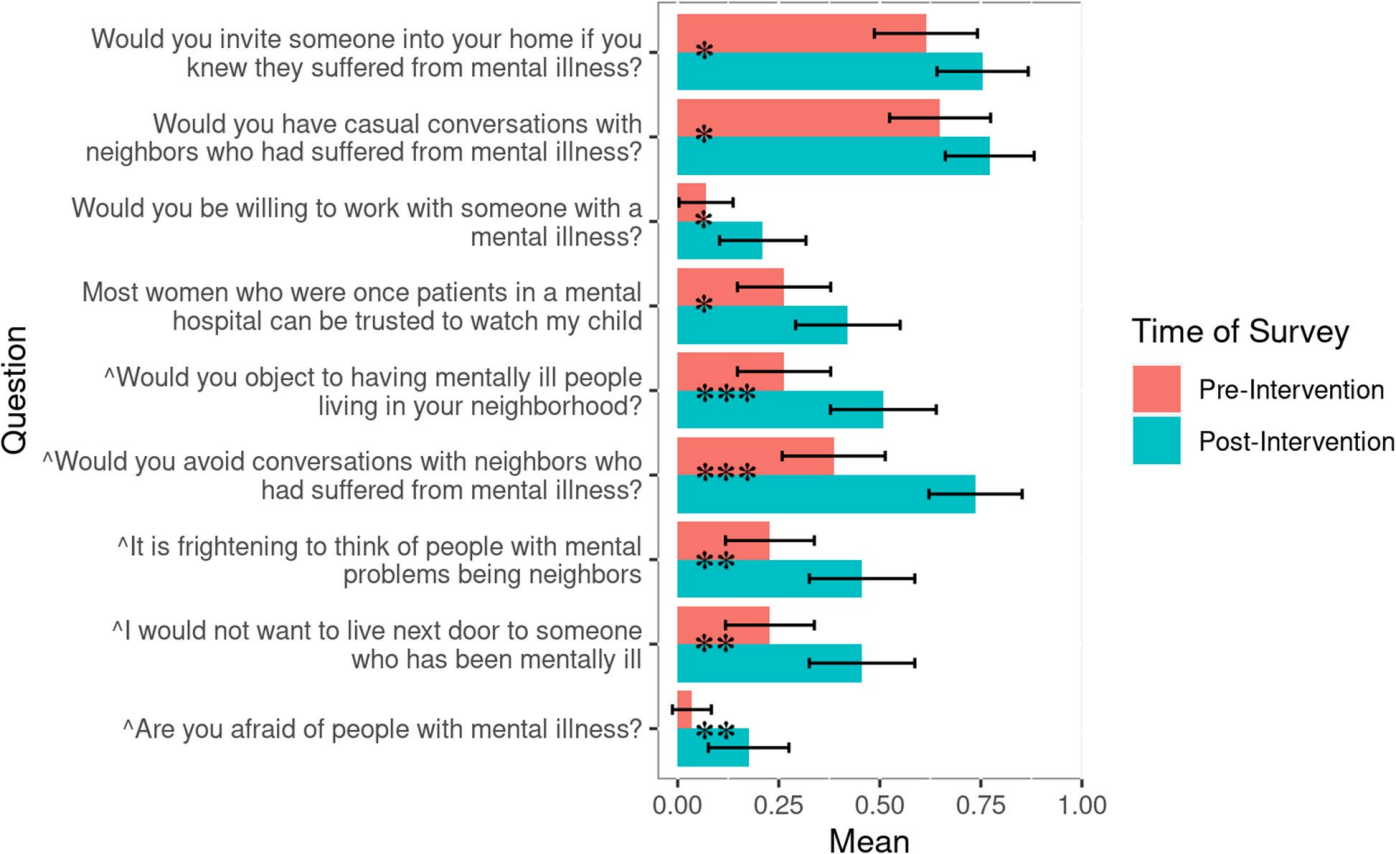
Average values from individual questions from the Personal Acceptance Scale before and after play intervention (n = 57). Questions were yes or no questions, corresponding to a 1 or 0 respectively. Some questions were reverse-scored so that increases in mean indicate greater personal acceptance. Error bars represent 95% confidence intervals. Statistical analysis in Appendix B, Table B2 ^reverse scored question. *p ≤ .05. **p ≤ .01. ***p ≤ .001

**Fig. 6:**
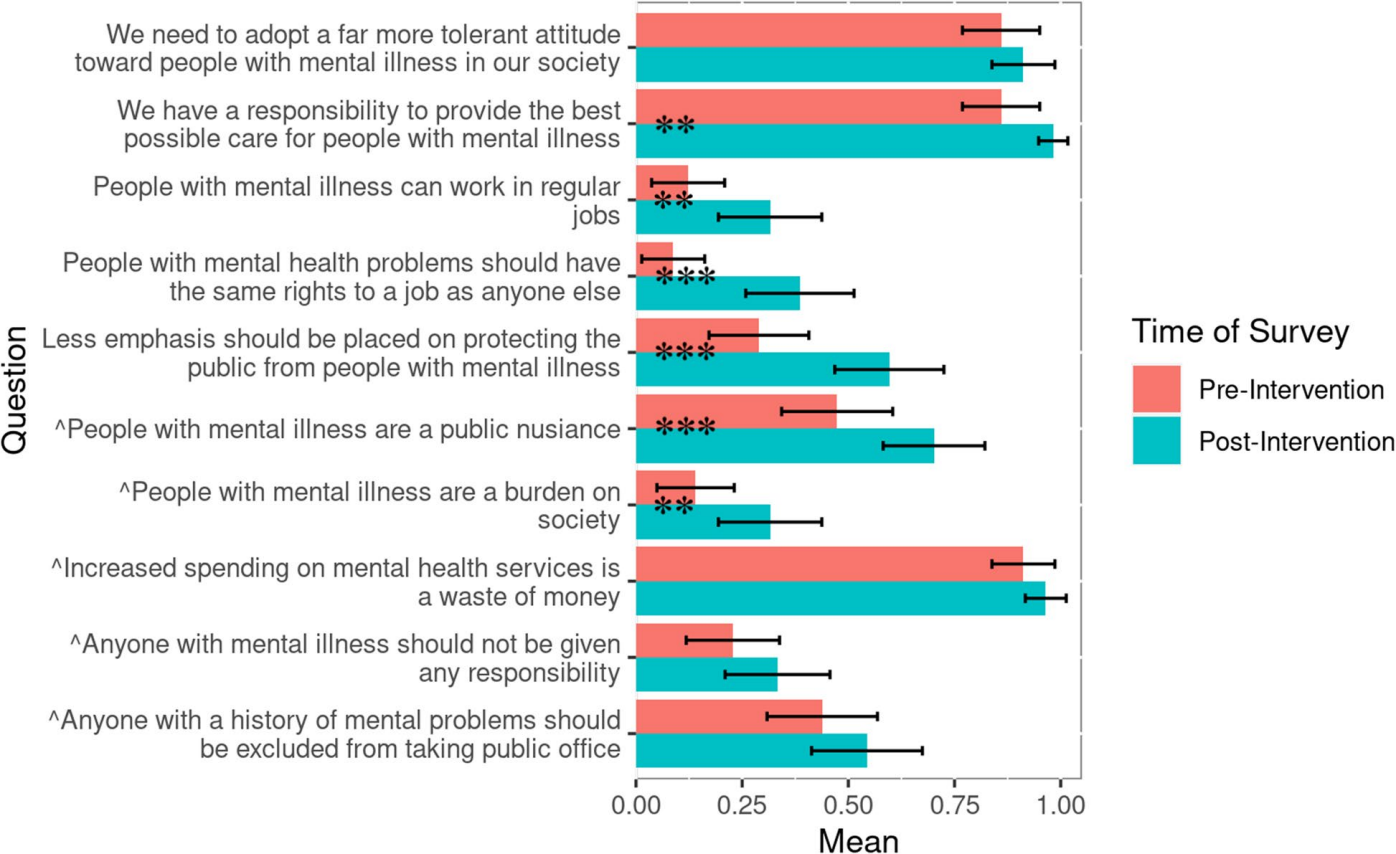
Average values from individual questions from the Broad Acceptance Scale before and after play intervention (n = 57). Questions were yes or no questions, corresponding to a 1 or 0. Some questions were reverse-scored so that increases in mean indicate greater broad acceptance. Error bars represent 95% confidence intervals. Statistical analysis in Appendix B, Table B2 ^reverse scored question. *p ≤ .05. **p ≤ .01. ***p ≤ .001

Individuals who viewed the theater performance reported a statistically significant increase in both personal acceptance and broad acceptance of individuals with mental illness/psychosis according to the Personal Acceptance Scale from pre-intervention responses (*M* = 3.04, *SD* = 2.12) to post-intervention responses (*M* = 4.99, *SD* = 2.74, *p* < .001, see Fig. 3), and the Broad Acceptance Scale from pre-intervention responses (*M* = 4.40, *SD* = 1.64) to post-intervention responses (*M* = 6.07, *SD* = 1.90, *p* < .001, see Fig. 4). Each individual question from the Personal Acceptance Scale and six out of ten questions from the Broad Acceptance Scale were statistically significant (*p* < .05) between pre-intervention and post-intervention responses (Figs. 5 and 6).

Post-intervention qualitative responses indicated more knowledge about psychiatric treatment and more favorable attitudes toward mentally ill patients. Most of the post-intervention cohort (93%) had a conversation about mental illness with another person after the play and 68% said they learned that mentally ill patients can fully recover with medical treatment (Table 2).

**Table 2:** Major response categories to open-ended post-intervention survey questions

**If you had a conversation about mental illness after seeing the play, with whom did you have the conversation?**^a^	**What was the conversation about?**^**c**^	**What did you learn from the play and training?**^**d**^
Relative(s) (61%)^b^	Patients can fully recover with medical treatment (45%)	Patients can fully recover with medical treatment (68%)
Neighbor(s) (36%)^b^	Patients should not be taken to a traditional healer/pastor (30%)	You should take patients to the health center (65%)
Friends(s) (18%)^b^	Summarized the play (28%)	Patients should not be taken to a traditional healer/pastor (35%)
Customers (2%)^b^	There is a clinician who can treat mental illness (21%)	Summarized the play (32%)
	Patients need community/family support (13%)	Patients need community/family support (21%)
	Causes, signs, symptoms (13%)	

^a^93% (53 people) reported having a conversation after seeing the play; only 44 people were asked with whom

^b^Number indicates percentage of participants that had a conversation with this party; some participants indicated multiple categories of people

^c^Question added just after the start of the survey, so not all participants were asked this question

^d^All respondents were asked this question

Responses to the focus group questions were sorted into five qualitative categories: causes of mental illness, signs and symptoms, treatment, post-treatment, and general comments. (Table 3). Causes of mental illness shared by participants included physical trauma, stress, alcohol and drugs, and family struggles. Signs and symptoms of mental illness included confusion, abnormal behaviors, violent behaviors, and physical changes. Treatment involved both modern and traditional medicine, and post-treatment indicated the possibility for people to fully recover, though if they did not, restraints and death were necessary for the protection of others. General comments indicated a belief that mental illness is not common in the community, a belief that those with mental illness are a burden to society, and a fear of mentally ill patients. These findings informed the criteria for the theater intervention.

**Table 3:** Synthesis of responses across all focus groups

Response Category^a^	Most Common Response and Themes^b^
*Causes*	●Physical damage to the brain, terminal physical illness ●Witchcraft (curses, ancestral spirits) ●Family and relationship struggles, domestic violence ●Stress, poverty, poor living conditions, alcohol/drug abuse
*Signs and Symptoms*	●Confusion and abnormal behavior: hallucinations, wandering far from home, disconnection from reality, running/walking around naked, eating from the trash, talking nonsense, restlessness ●Violent behavior: raping children, destruction of property, throwing stones at and beating others ●Physical changes: loss of appetite, headache, blurred vision, tearing eyes, nystagmus
*Treatment*	●Hospitals and modern medicine, prayer, and traditional healers are all valid initial treatments ●If the mental illness was caused by witchcraft, modern medicine will not be able to cure it, only traditional medicine will be effective ●Religion is recommended to people who are struggling with mental illness as a form of counseling ●Treatment for mental illness should include counseling and community support
*Post-Treatment*	●It is possible for people to recover fully from mental illnesses and return to their roles in society ●If treatment fails, people with mental illnesses should be tied up with rope/chains until death for the safety of themselves and others ●There should be a rehabilitation or isolation center (a protected place) where people can go if they remain untreated
*General Comments*	●Mental illness isn’t very common in our communities ●We are afraid of those with mental illnesses, even though not all are violent; we don’t want them as our neighbors; we don’t want to work with them ●Those with mental illness ○cannot work in regular jobs and shouldn’t be given responsibilities ○are a burden on society ○are dangerous because of violent behavior ●We as a community have a responsibility to care for people with mental illnesses; they deserve our sympathy ●We need to be more tolerant of those with mental illnesses

^a^Responses to the focus group questions were sorted into five qualitative categories based on participants’ open-ended responses. Prominent themes have been summarized for each area of discussion

^b^These findings informed the criteria for the theater intervention

## Discussion

### Statement of principal findings

The destigmatization community theater play followed by a brief verbal training showed a statistically significant decrease in participants’ stigma ratings on the Personal and Broad Acceptance Scales, showing that a community-led arts intervention, as previously used for HIV/AIDS, [28] can effectively be used to decrease the stigma of mental illness in a rural area of a low-income country. Viewing the play also led to decreased stigma and increased favorable attitudes towards psychiatric treatment, as evidenced by participants’ responses to qualitative questions regarding mental illness stigma. Most participants reported having conversations about the play with friends, family members, or neighbors, suggesting that the destigmatization could effectively spread beyond the direct audience of the play.

### Focus group findings

Focus group responses displayed a mix of traditional and modern scientific beliefs present in the surveyed population, from causes and treatments such as witchcraft to mentioning the use of hospitals (Table 3). The stigma surrounding mental illness was most often discussed in the general comments category of conversation as opposed to causes, signs and symptoms, treatment, or post-treatment; specifically, there was a demonstrated resistance to interacting with individuals suffering from mental illness (Table 3). This seemed to be rooted in concerns surrounding dangerous behaviors and the perceived threat towards the community that those suffering from mental illness were displaying, as one of the common signs of mental illness that community members brought up was violent behavior, including raping children, destroying property, throwing stones at people, and beating others (Table 3). Many individuals recognized that not all people suffering from mental illness were in fact violent (Table 3), but because of the small percentage with violent tendencies, there was a generalized fear. However, this fear of violence was balanced with a strong inclination toward empathy throughout the focus groups. This sentiment of empathy suggested that the community would be open to unlearning the stigma surrounding mental illness, a positive sign that the implementation of a well-designed theater play would achieve culturally sensitive and relevant destigmatization of mental illness.

### Strengths and weaknesses

Previous studies have emphasized the importance of community-based theater campaigns, citing their ability to connect with individuals emotionally and their basis in the credibility of community members, not outsiders [29. ]. These benefits are at the core of the theater intervention, and the aim of this study’s methodology was to utilize these principles for effective destigmatization. When researchers conduct an intervention by enforcing outside viewpoints without first gaining the trust of the community, the community is not as receptive and therefore does not experience as much destigmatization [30. ]. Community participation in the intervention empowers them to form and change their own body of knowledge [31]. In this study, local community health workers, who carry trust and respect within the community, were asked to generate their own skits that integrated common knowledge of the village with new beliefs and included traditional singing and dancing, as well as creative handcrafted props. The elected chairman of the village appeared at the plays and voiced support for the project and interventions, further establishing trust between researchers and community members. These community-based approaches made the productions more culturally relevant and enabled an effective intervention.

Language was a limitation of the study, as Lusoga does not have a word or phrase for mental illness in the same way that it is understood in English. Instead, the term for mental illness in Lusoga, *obulwaile bwakawanga*, roughly translates to “disease of the skull/brain”, which can reference any number of medical and mental conditions. Compounded by the fact that participants in rural villages have not encountered the phrase ‘mental illness’ when learning English in school, the incompatibility of language presents a rudimentary base to build from when trying to provide information, making it difficult to define mental illness. While the community health workers were given criteria for the play and a brief overview of the different types of mental illnesses, misconceptions about mental illness among the community health workers persisted. More debilitating and severe psychiatric disease, such as mania/psychosis, was depicted in the plays, but less obvious presentations, such as milder forms of depression and anxiety, were not included. Thus, the applicability of the project likely extends to the most debilitating mental illnesses, such as psychosis, mania, and severe anxiety or depression.

Additionally, brief verbal training conducted after the intervention helped clarify misunderstandings that could arise from the play. Although the exact contributions of the training to effective theater intervention is unclear, it was a component of the intervention and could have augmented the effects of the theater intervention. A weakness of the study is that we cannot measure how much this intervention contributed to destigmatization compared to the theater intervention as a stand-alone.

This study applies to rural Uganda, and further research is needed to determine effectiveness in urban regions of Uganda or areas of other low-income countries. Furthermore, the time between the intervention and the follow-up survey was approximately one week, and it is unclear if changes to stigma will persist over longer periods. Selection bias could have reduced generalizability to the population as participants were selected by household. Since women tended to be at home while their husbands were not in the household during participant recruitment, our participant pool was predominantly female. Unfortunately, the sample size was too small to assess whether or not this caused a bias in the results. However, gender distribution was similar between the baseline and intervention cohorts.

Several members of the baseline cohort did not attend the play or were not present for the follow-up survey. This dropout rate could have been due to a non-random reason, creating bias in the intervention cohort. However, a comparison of initial responses between the intervention cohort and the rest of the baseline cohort did not show a significant difference in attitudes in either the Personal Acceptance Scale or the Broad Acceptance Scale (Appendix C). Furthermore, there were no statistically significant differences in demographic information in regards to gender, education, and occupation between the two groups (Appendix B1). Some of the reasons for the dropout rate included participants traveling or occupied with their jobs when the play was shown or during the one-week period after the play when the post-intervention surveys were conducted. The dropout rate could have been improved by administering the pre-intervention surveys immediately before the intervention to participants attending the play.

#### Strengths and weakness in relation to other studies

To our knowledge, this was the first study evaluating the effectiveness of an arts-based intervention to decrease mental health stigma in a low-income country. There is overall a lack of research on stigma in low and middle-income countries, and there are even fewer studies evaluating the effectiveness of destigmatization interventions [32]. The interventions that have been conducted to reduce stigma have mainly been directed towards healthcare workers. One study in Nigeria found that a five-day direct training to healthcare workers improved perceptions of mental disorders and attitudes towards people with mental illness [33]. Another promising destigmatizing intervention has been integrating mental health into primary care in South Africa and Uganda. The authors found positive effects in attitudes of healthcare professionals [34].

Destigmatizing mental illness to health workers is critical, as they can treat or make referrals to appropriate facilities, but evidence shows that stigma in the community is a major barrier to seeking treatment [35, 36]. For example, in Colombia, Uribe et al. conducted a study related to help seeking that found that many individuals with mental illness did not disclose their status with anyone for fear of stigma [37]. There needs to be further exploration of strategies to reduce stigma towards mental illness at the community level, as it is one of the main contributors to not seeking mental health treatment globally [38].

#### Meaning of the study

Post-intervention survey responses showed more favorable attitudes towards living, working, and interacting with people with mental illness. Participants reported being less afraid of those with mental illness. Participants also reported a higher sense of community responsibility for treating and caring for those with mental illnesses, with significantly fewer individuals believing that mentally ill individuals are a burden or that spending on mental health services is a waste of money. These attitude changes may enable the success of future efforts to improve the provision and accessibility of mental health care and equitable treatment of those with mental illness, 2 and could indicate an increased willingness to support loved ones and other community members who are mentally ill, which is vital to their treatment [39].

The post-intervention survey also showed more favorable responses regarding the treatment of and recovery from mental illness, with a higher number of participants saying that patients should be treated at a hospital or health center and believing that individuals could fully recover from mental illness. There was also an increase in the prevalence of beliefs that people with mental illness have the right to a job, sympathy, and a “normal” life. An increase in these positive attitudes may encourage individuals with mental illness to seek treatment and may also support their integration into the community, [40] which could further reduce stigma within a community through the success of treatment [41, 42].

Of note, attitudes that contribute to structural stigma, reflected by the Broad Acceptance Scale, showed less of a significant reduction than public stigma, reflected by the Personal Acceptance Scale. For example, only six of ten items in the Broad Acceptance Scale showed a significant reduction compared with all of the items of the Personal Acceptance Scale. Although when taken together, Broad Acceptance Scale improved after the intervention significantly, this suggests that structural stigma may be more difficult to address with a community theater intervention. This finding is consistent with psychology literature that structural and public stigma are reduced through separate pathways, and our theater intervention centered around removing blame and humanizing those with mental illness, which is a pathway through which public stigma is reduced [43]. We did not address structural factors that lead to poor quality of life for those with mental illness in the intervention helping address why Broad Acceptance Scores did not improve as much as Personal Acceptance Scores. There is currently a dearth of studies that explore methods to reduce structural stigma, [44] and further work to explore more interventions that prioritize the reduction of structural stigma, which is a major contributor to inequity for those with mental illness, needs to be conducted [45].

Our survey and focus group responses show that there are significant misconceptions about mental illness among individuals living in these communities. Responses showed that individuals attribute multiple superstitious underlying causes to mental illness/psychosis afflictions, including possession by evil spirits, God’s punishment, and witchcraft (Appendix B, Table B3), consistent with prior studies in similar settings [46]. These beliefs did not change significantly between the pre- and post-intervention cohorts. This could be due to addressing causes of mental illness in the health professional’s training at the end of the performance, rather than in the play itself; the underlying biopsychosocial causes of mental illness/psychosis are also contradictory to pre-existing beliefs held by the community about mental illness, a point heavily emphasized in our focus group discussions. In contrast, the greatest decrease in stigma was observed in questions relating to community support and medical treatment. These topics were portrayed in the play itself and are more compatible with existing beliefs about the origins and presentation of mental illness. These results may indicate that interventions that add to fundamental beliefs are more effective than interventions that attempt to change them, and such interventions require nuanced understandings of community knowledge.

### Unanswered questions and future research

Following this study, further evaluation is necessary to see if there are lasting changes in stigma both among study participants and in the community at large, and if so, which changes are sustained. Additionally, more research is necessary to determine whether decreased stigma leads to an increase in care-seeking behaviors and if this type of intervention is equally effective against the stigma surrounding mental illnesses that do not often have as much of an obvious presentation, such as depression.

Nevertheless, theater-based interventions are a promising tool for destigmatization, and future work should build upon these strategies to effectively change attitudes about mental illness. Investing resources for mental health care not only in increased provision of care but also in community-led destigmatization campaigns would likely lead to improved community support and better outcomes for individuals suffering from mental illness. Furthermore, this type of arts intervention could be used outside of the Busoga region in rural areas of low-income countries with similar community structures, and it may be effective in destigmatizing conditions besides mental illness.

## Conclusion

The use of a community-led theater campaign for the destigmatization of mental health demonstrated a reduction in stigma. Positive changes were observed in participants’ beliefs and attitudes towards the rights and abilities of people with mental illness and the importance of medical treatment, potentially allowing people with mental illness to more easily seek treatment and remain connected to their community. This type of intervention could be expanded to other low-income countries with similar community structures, and it could be effective in use towards other stigmatized conditions. These findings may also indicate the advantages of investing mental health care resources in community-led campaigns for destigmatization as a part of improving mental health care.

## Supplementary Information

**Additional file 1.**

**Additional file 2.**

**Additional file 3: Appendix A.** **Table B1.** Participant information. **Table B2.** Responses to selectedquestions from the stigma survey. **Appendix B.** **Table B3.** Responses to causes of mentalillness. **Appendix C. Appendix D**.

## Data Availability

All data generated or analyzed during this study are included in this published article under supplementary material section.

## Declarations

Institutional Review Board - The AIDS Support Organization, Uganda: #TASOREC/048/2021-UG-REC-009.

Uganda National Council of Science and Technology registration number: HS1764ES.
