# Supplementary material for: Evaluation of a pilot, community-led mental illness de-stigmatization theater intervention in rural uganda
Source: BMC Psychiatry. 2022 Dec 16;22:794. doi: 10.1186/s12888-022-04441-w (PMC9756628; doi:10.1186/s12888-022-04441-w)
Supplement: Supplementary file 3 — Additional file 3: Appendix A. Table B1. Participant information. Table B2. Responses to selectedquestions from the stigma survey. Appendix B. Table B3. Responses to causes of mentalillness. Appendix C. Appendix D. [file 12888_2022_4441_MOESM3_ESM.docx]

**Appendix A**

In order to combat the issue of treating mental illnesses we have decided to ask you to compete in a competition where you are put into groups of up to 10 people and your groups will come up with a play. In these plays, we are hoping you can address some of the symptoms of mental illness and emphasize the effectiveness of medicine to treat them. We have come up with a list of criteria and examples of symptoms you can show in your play, but we are hoping for you to ultimately show that medicine can cure someone with a mental illness and that people with mental illness need to be respected, need to be loved and protected. We will watch the plays that you have come up with in about a week’s time and from there decide the winner who will receive a prize as well as put on their play for different groups. The winner will be performing their play many times so the prize is meant to be for your time commitment. Each play would be in the range of 5-20 minutes. Would you be interested in participating? If you would, please stick around and we will put you into groups based on location so that it is more convenient for you to get together with each other. We would be glad to answer any questions you might have.

After people who want to leave do so, we put them into groups and then go over the main topics and signs/symptoms. Check in with each group not this Thursday but next, and the final performance will be the following week.

- Check in with the groups (rehearsal), first 2 groups on Wednesday, second 2 groups on Thursday
- Play performances will be on the following Monday 7/26/2021

What to show in play:

1. Empathy

a. Focus on the needs of the patient

b. Not dangerous but need medicine to help them go back to normal

c. Not disabled, can be trusted with responsibilities and jobs

d. Can work and raise children

e. They need family support

f. Suicide and self-harm can be prevented; just need someone to talk to

2. Medical treatment and community support

a. All mental illnesses can be treated with medicine; full recovery is possible

b. Doesn’t have to impact cultural beliefs or religion, but work together to help the person get better

c. They need community support (like praying) as well as medicine in order to get better

d. It is an illness (like malaria or flu) but not contagious or a punishment from God

e. Anyone can have a mental illness, not a specific type of person

3. Different symptoms and mental illnesses

a. There are different signs and symptoms of the same mental illness

b. Manic symptoms are displayed externally

c. Depressive symptoms tend to be displayed internally

d. Try to include many different symptoms in the play rather than just one

Examples of signs and symptoms that can be used in play:

- Psychotic and manic symptoms

- Loss of concentration, confusion, disorganization, restlessness
- Eating from trash
- Seeing and hearing things that aren’t there
- Talking or laughing to themselves
- Recklessness and destructiveness
- Feeling good one day and then really bad the next: drastic change in behavior

- Depressive symptoms

- Always sad, tired, low motivation
- Feeling good one day and then really bad the next: drastic change in behavior
- Loss of appetite for food

**Appendix B**

**Table B1.** Participant information

| **Category** | | **Initial Number^c^** | **Final Number** |
| --- | --- | --- | --- |
| Gender | Male | 31 (30.7%) | 13  (22.8%) |
|  | Female | 70  (69.3%) | 44  (77.2%) |
| Age | | 40.2 ± 16.6 | 38.7 ± 15.8 |
| Religion | Anglican | 30  (29.7%) | 20  (35.1%) |
|  | Pentecostal | 22  (21.8%) | 17  (29.8%) |
|  | Catholic | 21  (20.8%) | 7  (12.3%) |
|  | Muslim | 22  (21.8%) | 10  (17.5%) |
|  | Other | 6  (5.9%) | 3  (5.3%) |
| Education | O-Level | 6  (5.9%) | 3  (5.3%) |
|  | Completed Primary | 29  (28.7%) | 17  (29.8%) |
|  | Incomplete Primary | 46  (45.5%) | 27  (47.4%) |
|  | Never Attended | 18  (17.8%) | 10  (17.5%) |
|  | Peasant farmer | 86  (85.1%) | 48  (84.2%) |
| Occupation | Other Work^a^ | 9  (8.9%) | 7  (12.3%) |
|  | Not Working^b^ | 6  (5.9%) | 2  (3.5%) |

^a^ Includes shop owner, tailor, and other occupations

^b^ Includes unemployed, students, and retired

^c^ Initial number represents the baseline cohort, and final number represents those in the baseline cohort that watched the theater intervention and were able to be found for the follow-up questionnaire

**Table B2.** Responses to selected questions from the stigma survey.

| **Question^a^** | **Mean Initial ± SD** | **Mean Final ± SD** | **Statistics** |
| --- | --- | --- | --- |
| ^b^ People with mental illness are a public nuisance | .47 ± .50 | .70 ± .46 | *t* (56) = 2.88, *p* < .001***, *d* = 0.47 |
| People with mental illness can work in regular jobs | .12 ± .33 | .32 ± .47 | *t* (56) = 2.83, *p* = .01**, *d* = 0.48 |
| ^b^ Anyone with mental illness should not be given any responsibility | .23 ± .42 | .33 ± .48 | *t* (56) = 1.43, *p* = .16, *d* = 0.23 |
| ^b^ Anyone with a history of mental problems should be excluded from taking public office. | .44 ± .50 | .54 ± .50 | *t* (56) = 1.29, *p* = .20, *d* = 0.21 |
| ^b^ People with mental illness are a burden on society | .14 ± .35 | .32 ± .47 | *t* (56) = 2.83, *p* = .01**, *d* = 0.42 |
| We have a responsibility to provide the best possible care for people with mental illness | .86 ± .35 | .98 ± .13 | *t* (56) = 2.80, *p* = .01**, *d* = 0.46 |
| ^b^ Increased spending on mental health services is a waste of money | .91 ± .29 | .96 ± .19 | *t* (56) = 1.35, *p* = .18, *d* = 0.22 |
| We need to adopt a far more tolerant attitude toward people with mental illness in our society | .86 ± .35 | .91 ± .29 | *t* (56) = 0.90, *p* = .37, *d* = 0.16 |
| Less emphasis should be placed on protecting the public from people with mental illness | .29 ± .45 | .60 ± .49 | *t* (56) = 3.71, *p* < .001***, *d* = 0.65 |
| People with mental health problems should have the same rights to a job as anyone else | .09 ± .29 | .39 ± .49 | *t* (56) = 4.51, *p* < .001***, *d* = 0.74 |
| ^b^ Are you afraid of people with mental illness? | .04 ± .19 | .18 ± .18 | *t* (56) = 2.66, *p* = .01**, *d* = 0.47 |
| ^b^ Would you object to having mentally ill people living in your neighborhood? | .26 ± .44 | .51 ± .50 | *t* (56) = 3.64, *p* < .001***, *d* = 0.52 |
| ^b^ Would you avoid conversations with neighbors who had suffered from mental illness? | .39 ± .49 | .74 ± .44 | *t* (56) = 4.81, *p* < .001***, *d* = 0.75 |
| Would you be willing to work with someone with a mental illness? | .07 ± .26 | .21 ± .41 | *t* (56) = 2.21, *p* = .03*, *d* = 0.41 |
| Would you invite someone into your home if you knew they suffered from mental illness? | .61 ± .49 | .75 ± .43 | *t* (56) = 2.06, *p* = .04*, *d* = 0.30 |
| Would you have casual conversations with neighbors who had suffered from mental illness? | .65 ± .48 | .77 ± .42 | *t* (56) = 1.99, *p* = .05*, *d* = 0.27 |
| ^b^ It is frightening to think of people with mental problems being neighbors | .23 ± .42 | .46 ± .50 | *t* (56) = 2.88, *p* = .006**, *d* = 0.49 |
| ^b^ I would not want to live next door to someone who has been mentally ill | .23 ± .42 | .46 ± .50 | *t* (56) = 2.88, *p* = .006**, *d* = 0.49 |
| Most women who were once patients in a mental hospital can be trusted to watch my child | .26 ± .44 | .42 ± .50 | *t* (56) = 2.42, *p* = .02*, *d* = 0.33 |

^a^ Questions chosen because of usage in scales along with general importance in analysis of attitudes. Questions were yes/no questions and converted to either 1 or 0. Only data from participants who filled out both pre-intervention and post-intervention surveys was included (n = 57).

^b^ reverse scored here and for the scales.

**p* ≤ .05

***p* ≤ .01

****p* ≤ .001

**Table B3.** Responses to causes of mental illness

| **Which of the following can cause mental illness?^a^** | **Mean Initial ± SD** | **Mean Final ± SD** | **Statistics** |
| --- | --- | --- | --- |
| Drug or alcohol misuse | .90 ± .27 | .95 ± .23 | *t*(56) = 0.90, *p* < .37, *d* = 0.17 |
| Possession by evil spirits | .79 ± .40 | .86 ± .35 | *t*(56) = 1.05, *p* = .30, *d* = 0.19 |
| Traumatic event or shock | .64 ± .48 | .86 ± .35 | *t*(56) = 2.81, *p* = .01**, *d* = 0.52 |
| Stress | .88 ± .33 | .98 ± .13 | *t*(56) = 2.57, *p* = .01**, *d* = 0.42 |
| Genetic inheritance | .48 ± .49 | .53 ± .50 | *t*(56) = 0.47, *p* = .64, *d* = 0.09 |
| Physical abuse | .66 ± .47 | .31 ± .59 | *t*(56) = 3.70, *p* < .001***, *d* = 0.46 |
| Biological factors (other than brain disease or genetics) | .75 ± .41 | .82 ± .37 | *t*(56) = 1.29, *p* = .20, *d* = 0.20 |
| God’s punishment | .67 ± .47 | .61 ± .49 | *t*(56) = 0.73, *p* = .47, *d* = 0.13 |
| Witchcraft | .95 ± .23 | .88 ± .33 | *t*(56) = 1.43 *p* < .16, *d* = 0.25 |
| Brain disease | .67 ± .47 | .61 ± .49 | *t*(56) = 0.73, *p* < .47, *d* = 0.13 |
| Poverty | .61 ± .49 | .77 ± .42 | *t*(56) = 2.02, *p* = .05*, *d* = 3.44 |

^a^ Questions were yes/no questions and converted to either 1 or 0. Only data from participants who filled out both pre-intervention and post-intervention surveys was included (n = 57).

**p* ≤ .05

***p* ≤ .01

****p* ≤ .001

**Appendix C**

According to an independent samples t-test comparing initial responses on the Personal Acceptance Scale, which evaluated personal attitudes and stigma towards mental health, between participants who were resurveyed (*M* = 3.04, *SD* = 2.12) and participants who were excluded (*M* = 3.01, *SD* = 2.08), there was no significant difference, *t*(99) = 0.09, *p* = .93. On the Broad Acceptance Scale, which evaluated community attitudes and stigma towards mental health, participants who were resurveyed (*M* = 4.41, *SD* = 1.62) had similar attitudes compared to participants who were excluded (*M* = 4.53, *SD* = 1.45), *t*(99) = 0.39, *p* = .70.

**Appendix D.** Codebook for focus group discussions

| **Themes** | **Sub-Themes** | | **Illustrative Quotes** |
| --- | --- | --- | --- |
| Manifestations of mental illness | Causes | Medicalized | “…it can start with cerebral Malaria and then…that person has mental illness...” (FG2, Female) |
|  |  | Spiritual | “…mental illness can originate from witchcraft…” (FG4, Male) |
|  | Symptoms | Disoriented | “…walk long distances away from their homes; when the person gets back to normal senses, they will not remember how they made it to wherever they were.” (FG3, Female) |
|  |  | Dangerous | “…they can beat or throw stones at you.”(FG3, Female) |
| Treatment methodology | Western medicine | | “…not all the mental illnesses are the same, we need to take them to the hospitals so that doctors can diagnose and also give treatments.”(FG3, Female) |
|  | Traditional medicine | | “I think such people cannot be treated with drugs especially if it was caused by witchcraft, and I am convinced that it can treated by spiritual healers or traditional healers.” (FG1, Male) |
| Perception of those with mental illness | Fearful | | “I would tie him with a rope or chains to prevent him or her from escaping or beating other people or breaking people’s things or harming children.”(FG3, Female) |
|  | Empathetic | | “I do believe that mentally ill people are also human like us that need care and friendship and sympathy like we do.” (FG1, Male) |
